# C1q/TNF‐Related Protein 4 (C1QTNF4) Acts as an Adipokine That Ameliorates Diet‐Induced Obesity by Improving Energy Metabolism and Alleviating Adipose Inflammation

**DOI:** 10.1155/mi/7613074

**Published:** 2026-05-09

**Authors:** Daxiang Na, Yanwei He, Xinrui Liu, Mingjun Huang, Shuo Xie, Hexin Li, Jingying Liu, Hong Zhang, Lu Wang

**Affiliations:** ^1^ School of Basic Medical Sciences, Peking University Health Science Center, Peking University, Beijing, 100191, China, pku.edu.cn; ^2^ Department of Immunology, Key Laboratory of Medical Immunology, Ministry of Health, Peking University, Beijing, 100191, China, pku.edu.cn; ^3^ State Key Laboratory of Vascular Homeostasis and Remodeling, The Institute of Cardiovascular Sciences, Peking University, Beijing, 100191, China, pku.edu.cn; ^4^ Biological Sample Management Center, Beijing Hospital, Beijing, 100730, China, bjhmoh.cn; ^5^ Department of Cardiology, Fuwai Hospital, Chinese Academy of Medical Sciences & Peking Union Medical College/National Center for Cardiovascular Diseases, Beijing, 100037, China, fuwaihospital.org

**Keywords:** C1QTNF4, inflammation, metabolic disease, obesity

## Abstract

**Background:**

The C1q/TNF‐related protein (C1QTNF) plays a crucial role in the intricate connection between obesity and inflammation, acting as a significant adipokine. This study aims to investigate the potential of C1QTNF4 as an antiobesity and anti‐inflammatory adipokine.

**Methods:**

Forty‐eight subjects, with or without obesity and diabetes mellitus (DM), were categorized into two groups based on their health condition. Measurements of C1QTNF4, glucose, and adipokine concentrations were conducted. Seven‐week‐old C57BL/6J mice were segregated into four groups, with eight mice in each group of both sexes. C1QTNF4 transgenic (Tg) mice and their corresponding littermate controls were subjected to either a high‐fat (HF) diet (HFD) or a standard chow diet for 14 weeks.

**Results:**

C1QTNF4 and leptin levels increased, while adiponectin levels decreased in subjects with obesity and DM compared to normal individuals. C1QTNF4 Tg mice on a HFD exhibited resistance to weight gain, accompanied by an amelioration of insulin resistance (IR). Fatty liver and chronic adipose inflammation were also mitigated. In the liver and skeletal muscle of C1QTNF4 Tg mice, C1QTNF4 activated the AMP‐activated protein kinase (AMPK) pathway associated with insulin sensitivity and energy expenditure. Plasma IL‐6 decreased in Tg mice on a HFD, indicating that C1QTNF4 alleviates inflammation via the IL‐6‐JAK1/2‐STAT3 pathway.

**Conclusions:**

C1QTNF4 emerges as an adipokine with a regulatory role in adipogenesis, addressing energy imbalances and reducing inflammation. This study suggests that C1QTNF4 represents a potential drug target for treating obesity, IR, and inflammation.

## 1. Introduction

As global health epidemics, obesity and its pathological consequences, such as type 2 diabetes and cardiovascular diseases, have received increasing attention in recent years [1]. According to a 2024 study by the NCD Risk Factor Collaboration (NCD‐RisC) published in The Lancet, ~890 million adults worldwide were living with obesity (body mass index [BMI] ≥ 30) in 2022, with an age‐standardized prevalence of ~16%. Furthermore, an analysis based on the Global Burden of Disease Study 2021, published in EClinicalMedicine in 2024, revealed that the health burden attributable to high body mass index (BMI > 25 kg/m^2^) remains substantial, causing a global age‐standardized death rate (ASDR) of ~44.23 per 100,000 population in 2021 [2, 3]. Such interest has resulted in extensive studies on adipose tissue, which has been characterized as an important regulator of metabolic homeostasis. In the context of chronic inflammation, several proinflammatory cytokines produced by immune and nonimmune cells are widely thought to be significant promoters of obesity.

In addition to serving as an energy storage tissue, adipose tissue has been recognized as an endocrine organ ever since the discovery of leptin. The proteins secreted by adipose tissue are termed “adipokines.” While many adipokines function as factors that promote obesity and regulate inflammation, some play protective roles against metabolic disorders and possess anti‐inflammatory properties, exemplified by adiponectin and leptin [4]. AMP‐activated protein kinase (AMPK) is a threonine/serine kinase crucial for energy metabolism and is considered a cellular “fuel gauge” and redox sensor. Accumulating evidence indicates that inhibiting AMPK leads to insulin resistance (IR), whereas activating AMPK enhances insulin sensitivity [5].

Adiponectin stands out as a well‐studied adipokine, renowned for its extensively described antiobesity, antidiabetic, and anti‐inflammatory functions [6–9]. In recent years, the C1q/TNF‐related protein (C1QTNF) family members, homologs of adiponectin, have become research hotspots [10]. These family members share a common structural framework, featuring a signal peptide in the N terminus, a short variable domain, a collagen‐like domain in the middle, and a highly conserved Cq domain at the C terminus [11]. However, C1QTNF4 deviates from this pattern within the family, exhibiting two Cq globular domains and lacking a collagen‐like domain. The functions of C1QTNF family members encompass a broad spectrum, ranging from influencing glucose metabolism [12] to regulating inflammation [10] and participating in fatty acid oxidation [13]. Consequently, the C1QTNF family is considered a pivotal link connecting obesity and inflammation. It is widely thought that in obesity, C1QTNF1 may potentially compensate for IR, while C1QTNF3 emerges as a potent anti‐inflammatory adipokine, inhibiting proinflammatory pathways [14]. Human C1QTNF4, a member of the C1QTNF family, was originally identified by Wong et al. [11]. This highly conserved family consists of secreted proteins, initially cloned based on an adiponectin homology sequence [11], and has demonstrated close associations with immune inflammation and metabolism [14]. C1QTNF3, derived from adipocytes and mesenteric adipose tissue, attenuates MIF, MCP‐1, CCL4, and downregulates LPS‐induced proinflammatory signaling. C1QTNF6, predominantly expressed in adipose tissue, augments anti‐inflammatory IL‐10 in macrophages via p42/44 MAPK signaling and activates AMPK to promote fatty acid oxidation, regulating metabolism in obesity. C1QTNF9, an adipose‐specific adipokine sharing the AdipoR1 receptor with adiponectin, enhances endothelial vasorelaxation via AdipoR1/AMPK/eNOS signaling, prevents vascular smooth muscle proliferation, and activates AMPK and Akt to increase insulin‐mediated glucose uptake, attenuating IR and hepatic steatosis. C1QTNF12 (adipolin) improves insulin sensitivity and glucose tolerance in obesity, is regulated by KLF transcription factors, and attenuates proinflammatory macrophage infiltration and cytokine expression via Akt signaling, thereby downregulating gluconeogenesis and enhancing glucose uptake [15]. In our earlier research, we first characterized C1QTNF4 and observed its role in promoting the survival and proliferation of cancer cells [16]. A recent study highlighted C1QTNF4’s ability to suppress food intake behavior within 3 days through the regulation of the central nervous system [17]. Furthermore, another recent study underscored the sex‐dependent physiological role of C1QTNF4 in modulating food intake patterns and systemic energy metabolism [18]. Recent studies have revealed that C1QTNF4 binds to IL6R, thereby regulating the STAT3 pathway. As such, C1QTNF4 could potentially serve as a therapeutic intervention for Th17‐driven autoimmune diseases [19]. Additionally, our recent findings revealed that C1QTNF4 inhibits vascular smooth muscle cell proliferation and migration, thereby mitigating vascular remodeling [20]. Given its distinctiveness within the novel adipokine family, we posit that the functions of C1QTNF4 extend beyond those previously investigated.

This study presents a comprehensive exploration of the metabolic and inflammatory impacts of C1QTNF4, revealing its multifaceted functions. C1QTNF4 exhibits a repressive role in adipose metabolism. The metabolic profiles observed in C1QTNF4 transgenic (Tg) mice suggest that C1QTNF4 confers favorable effects on energy imbalances through modulation of the AMPK/ACC and AMPK/S6K1/sterol regulatory element‐binding protein 1c (Srebp1c) pathways in skeletal muscle and liver tissues. Moreover, C1QTNF4 demonstrates inhibitory effects on IL‐6 and its downstream pathways within the Tg model. Collectively, based on this compelling evidence, we posit that C1QTNF4 functions as an antiobesity and anti‐inflammatory adipokine, intricately regulating metabolism, and inflammation.

## 2. Results

### 2.1. Altered Expression of C1QTNF4 in Organisms With Energy Imbalance

**Figure 1:**
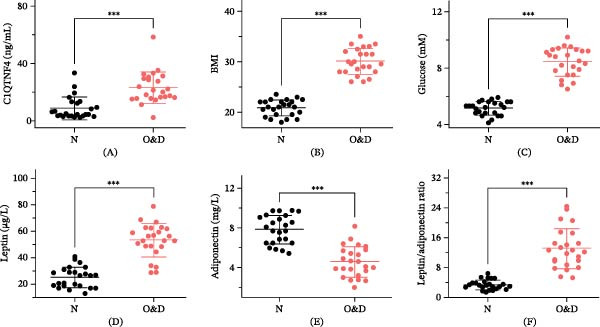
BMI, plasma C1QTNF4, glucose, leptin, and adiponectin levels, and leptin/adiponectin ratio in normal and diabetic individuals—48 subjects of both sexes. “N” denotes normal individuals; “O&D” represents obese and diabetic individuals. (A) C1QTNF4 levels in normal and obese/diabetic individuals. (B) BMI of the two groups of individuals. (C–F) Blood glucose, leptin levels, adiponectin levels, and leptin/adiponectin ratios in different groups of individuals.

To investigate the potential influence of whole‐body energy balance alterations on C1QTNF4 expression, we conducted a comparative study of plasma C1QTNF4 levels in healthy individuals and those with both obesity and diabetes. The results revealed a statistically significant elevation in C1QTNF4 levels within the obese/diabetic group (23.01 ± 2.256 ng/mL) compared to the healthy control group (8.639 ± 1.605 ng/mL) (Figure 1A). The BMI of the obese/diabetic group ranged from 28 to 36 kg/m^2^ (Figure 1B). This finding suggests a potential link between metabolic stress and C1QTNF4 expression. Further analysis revealed no significant differences in C1QTNF4 expression based on gender in the healthy group (Figure S1A), whereas in the obesity/diabetes group, there were no significant difference in the expression of C1QTNF4 by gender (Figure S1B). However, statistically significant differences were observed when comparing C1QTNF4 levels between healthy and obese/diabetic males (Figure S1C) and between healthy and obese/diabetic females (Figure S1D). Additionally, the study identified significant increases in circulating glucose (8.438 ± 0.2059 mM vs. 5.150 ± 0.1000 mM), leptin (53.05 ± 2.576 μg/L vs. 24.98 ± 1.588 μg/L), and the leptin/adiponectin ratio (12.99 ± 1.100 vs. 3.340 ± 0.2636) in obese/diabetic individuals compared to healthy individuals (Figure 1C–F). Conversely, adiponectin levels exhibited a contrasting pattern, with a significant decrease observed in obese/diabetic patients compared to healthy controls (7.809 ± 0.2950 mg/L vs. 4.558 ± 0.3171 mg/L). These findings collectively suggest that C1QTNF4 and leptin expression are elevated in obese and diabetic individuals compared to healthy subjects, while adiponectin expression is reduced, leading to a concomitant leptin/adiponectin imbalance.

### 2.2. C1QTNF4 Tg Mice Showed Resistance to Challenge With a High‐Fat (HF) Diet (HFD)

**Figure 2:**
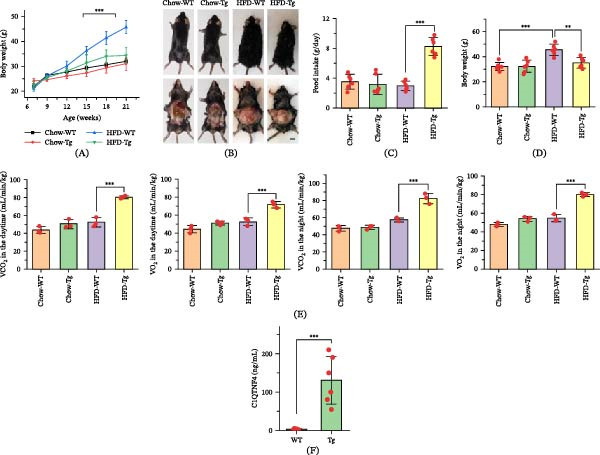
Diet‐induced obesity in WT and C1QTNF4 Tg mice. (A) Body weights of WT and C1QTNF4 Tg mice on chow or a high‐fat (HF) diet. (B) Photographs taken during mouse necropsies with a scale bar of 1 cm. (C) Daily food intake of the mice over 18 weeks. (D) The average mouse body weight measured during the 21st week. (E) Peak O_2_ consumption at exhaustion (VO2) and CO_2_ exhalation (VCO_2_) during daytime and nighttime treadmill testing for mice on the chow and HF diet. (F) Serum levels of human C1QTNF4 protein in mice fed a chow diet. The data are presented as the mean ± SD for 8 mice/group.  ^∗∗^, *p* ≤ 0.01;  ^∗∗∗^, *p* ≤ 0.001.

Mice demonstrated high sequence homology in C1QTNF4 with humans, exceeding 99% (Figure S2A–C). This observation prompted the hypothesis that Tg mice could serve as a valuable model to investigate the functional role of C1QTNF4. Initial investigations, however, did not reveal any significant differences in body weight between Tg and wild‐type (WT) control mice under standard dietary conditions (Figure 2A,D). Additionally, during treadmill exercise tests, Tg and WT mice exhibited comparable VO_2_ and VCO_2_ values, both during the day and night (Figure 2E). These initial findings suggested a minimal impact of C1QTNF4 overexpression on basal energy expenditure under standard conditions. Subsequently, a diet‐induced obesity (DIO) model was established using C1QTNF4 Tg mice. Compared to WT control mice (2.167 ± 0.6009 ng/mL), serum C1QTNF4 levels in the Tg group were significantly elevated (130.8 ± 25.44 ng/mL) (Figure 2F). Notably, despite consuming 220% more food than WT mice, Tg mice displayed a paradoxical 23% reduction in body weight after 14 weeks on a HFD (Figure 2A–D). The fat mass, lean mass, and their proportions relative to body weight in Tg mice exhibited similar changes to those observed in the aforementioned body weight variations (Figure S2D–G). Interestingly, Tg mice exhibited significantly higher VO_2_ and VCO_2_ values compared to WT controls during both daytime and nighttime measurements (Figure 2E). These findings indicate that Tg mice expended a greater amount of energy compared to their WT counterparts, potentially explaining their increased food intake yet paradoxically lower body weight under HFD conditions.

### 2.3. C1QTNF4 Tg Mice Showed High Sensitivity to Insulin

**Figure 3:**
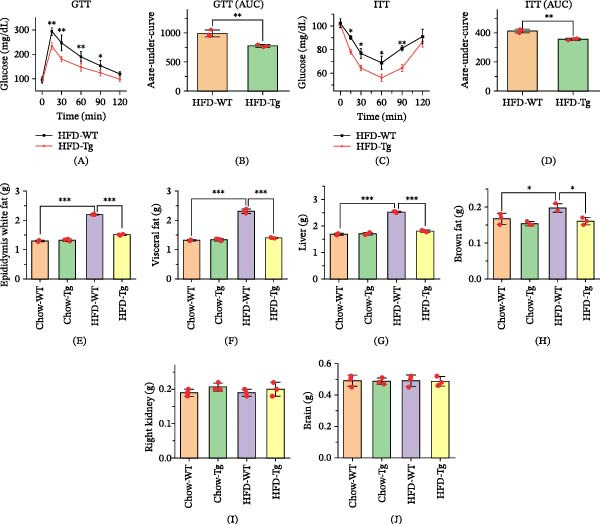
Insulin sensitivity and tissue weights of C1QTNF4 transgenic mice. (A) Glucose tolerance test (GTT) results for mice on high‐fat (HF) diets. (B) Quantification of cumulative glucose clearance during the GTT by integration of the area under the curve (AUC). (C) Insulin tolerance test (ITT) results for mice on HF diets. (D) Quantification of cumulative glucose clearance during the ITT by integration of the AUC. (E–J) Weights of epididymal white fat, visceral fat, liver, brown fat, right kidney, and brain in HF and chow diet‐fed mice. The data are presented as the mean ± SD for 8 mice/group.  ^∗^, *p* ≤ 0.05;  ^∗∗^, *p* ≤ 0.01;  ^∗∗^, *p* ≤ 0.001.

Following intraperitoneal glucose injection, Tg mice on an HFD exhibited significantly slower blood glucose rise compared to WT mice. Additionally, Tg mice demonstrated faster glucose clearance, as evidenced by both Figure 3A and the calculated area under the curve (AUC) in Figure 3B. This enhanced cumulative glucose disposal indicated greater insulin sensitivity in Tg mice. This finding was further corroborated by the insulin tolerance test results (Figure 3C,D), where Tg mice displayed superior cumulative glucose clearance in response to insulin administration compared to HFD‐fed WT mice. Notably, visceral fat, liver, and brown fat weights were all significantly reduced in Tg mice compared to WT mice on the HF. The weights of the right kidney and brain did not change in four groups. This pattern extended to epididymal fat, specifically in male Tg mice compared to their male WT counterparts (Figure 3E–J).

### 2.4. Adipose Tissue Inflammation Was Ameliorated in C1QTNF4 Tg Mice, and HFD‐Fed Tg Mice Had Lipid Metabolism Abnormalities in the Liver

**Figure 4:**
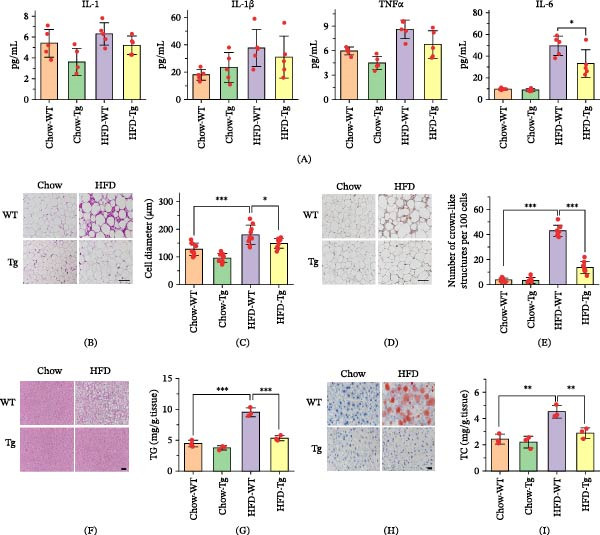
Inflammatory milieu in C1QTNF4 Tg mice. (A) Plasma cytokine levels of wild‐type (WT) and Tg mice on chow or HF diets. (B) Hematoxylin and eosin (H&E) staining of epididymal white adipose tissue (WAT) in mice. Scale bar, 100 μm. (C) Quantitative measurements of cell diameters. (D) Immunohistochemical staining of F4/80 in epididymal WAT. Scale bar, 100 μm. (E) Quantitative measurements of the numbers of crown‐like structures (CLSs). (F) H&E staining of a liver section. Scale bar, 100 μm. (G) Quantitative measurements of triglyceride (TG) levels in WT and Tg mice on HF and chow diets. (H) Oil Red O staining of a liver section. Scale bar, 50 μm. (I) Quantitative measurements of total cholesterol (TC) levels in WT and Tg mice on HF and chow diets. The data are presented as the mean ± SD for 8 mice/group.  ^∗^, *p* ≤ 0.05;  ^∗∗^, *p* ≤ 0.001;  ^∗∗∗^, *p* ≤ 0.0001.

Following previous research implicating C1QTNF4 in anti‐inflammatory processes [20], the present study investigated its potential to mitigate inflammation induced by an HFD in mice. Cytokine profiles in the plasma of both healthy and obese animals were analyzed, focusing on IL‐6, IL‐1, IL‐10, and TNFα. While the HFD elevated all four cytokines, subsequent administration of C1QTNF4 via transgenesis demonstrated significant suppression of IL‐6 levels in Tg mice compared to WT controls (16.40 ± 4.686 pg/mL vs. control). Although decreasing trends of the other three cytokines were observed in Tg mice, these changes did not reach statistical significance (Figure 4A). Histological analysis further supported the anti‐inflammatory effect of C1QTNF4. Hematoxylin and eosin (H&E) staining revealed that Tg mice exhibited substantially less enlargement of white adipose tissue (WAT) cells compared to WT mice on the HFD (Figure 4B). Additionally, immunohistochemical staining with an anti‐F4/80 antibody indicated a striking 77.5% reduction in crown‐like structure (CLS) infiltration in the WAT of Tg mice, indicative of diminished inflammation (Figure 4C,D). Hepatic histology, assessed by H&E and Oil Red O staining, concurred with these findings, demonstrating a significant reduction in the number and size of lipid droplets in the livers of Tg mice compared to WT controls under HFD conditions (Figure 4E,F). However, analysis of liver lipid metabolism parameters revealed persistent abnormalities in Tg mice on the HFD, suggesting incomplete metabolic recovery despite reduced inflammation. Nevertheless, compared to WT animals on the HFD, Tg mice displayed lower levels of both triglycerides (TGs) and total cholesterol (TC), indicating a trend toward improved lipid homeostasis (Figure 4G–I).

### 2.5. The AMPK‐ACC, AMPK‐S6K1‐Srebp1c, and JAK/STAT3 Pathways Were Activated by C1QTNF4

**Figure 5:**
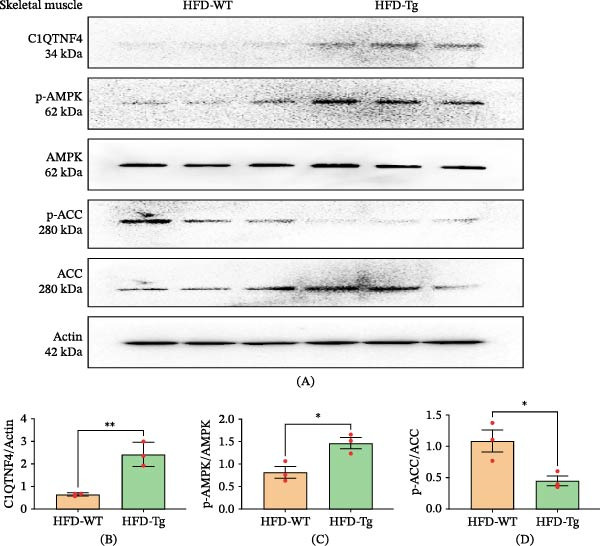
Western blot results for skeletal muscle. (A) C1QTNF4, AMPK, ACC, and actin signaling in the muscle tissue of WT and Tg mice on an HF diet. (B–D) Quantitative measurements of C1QTNF4, pAMPK/AMPK, and pACC/ACC.

**Figure 6:**
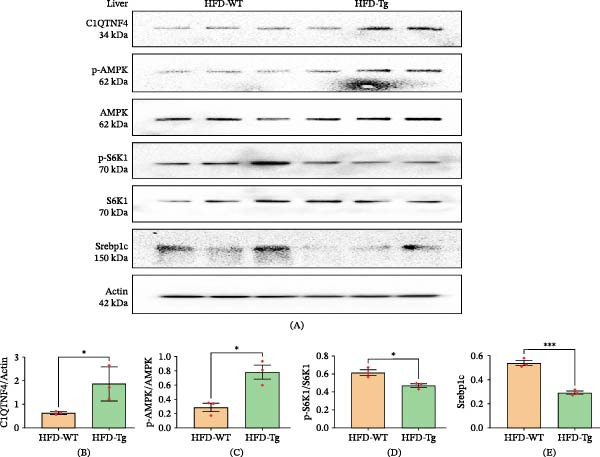
Western blot results for the liver. (A) C1QTNF4, AMPK, S6K1, Srebp1c, and actin signaling in the livers of WT and Tg mice on an HF diet. (B–E) Quantitative measurements of C1QTNF4, pAMPK/AMPK, pS6K1/S6K1, and Srebp1c.

**Figure 7:**
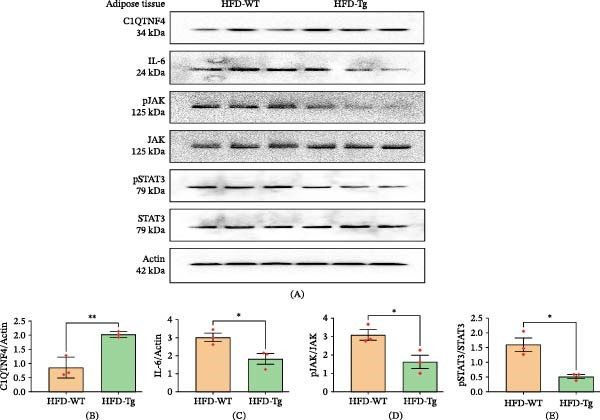
Western blot results for adipose tissue. (A) C1QTNF4, IL‐6, JAK, STAT3, and actin signaling in the adipose tissue of WT and Tg mice on the HF diet. (B–E) Quantitative measurements of C1QTNF4, IL‐6, pJAK/JAK, and pSTAT3/STAT3.

In a Tg mouse model overexpressing C1QTNF4 (C1QTNF4 Tg mice), compared to WT mice, significantly higher levels of C1QTNF4 were detected. This elevated expression was accompanied by activation of the AMPK pathway, as evidenced by phosphorylation of AMPKα at Thr‐172. Western blot analysis further confirmed this activation, revealing increased levels of phosphorylated AMPK (pAMPK) and decreased levels of phosphorylated acetyl‐CoA carboxylase (pACC) in skeletal muscle. Similarly, in the liver, elevated pAMPK was observed alongside the downregulation of phosphorylated S6 kinase 1 (pS6K1) and Srebp1c (Figures 5 and 6). Interestingly, IL‐6 levels were found to be elevated in mice fed an HFD compared to those on a chow diet. Notably, HFD‐fed C1QTNF4 Tg mice exhibited lower IL‐6 levels compared to their WT counterparts. Additionally, reduced phosphorylated JAK and STAT3 protein levels were observed in adipose tissue of C1QTNF4 Tg mice, suggesting a potential role for C1QTNF4 in suppressing inflammation via the JAK/STAT3 pathway (Figure 7). The changes in protein expression observed by Western blot were validated at the mRNA level by RT‐qPCR. These results were similar to Western blot (Figure S3).

## 3. Discussion

**Figure 8:**
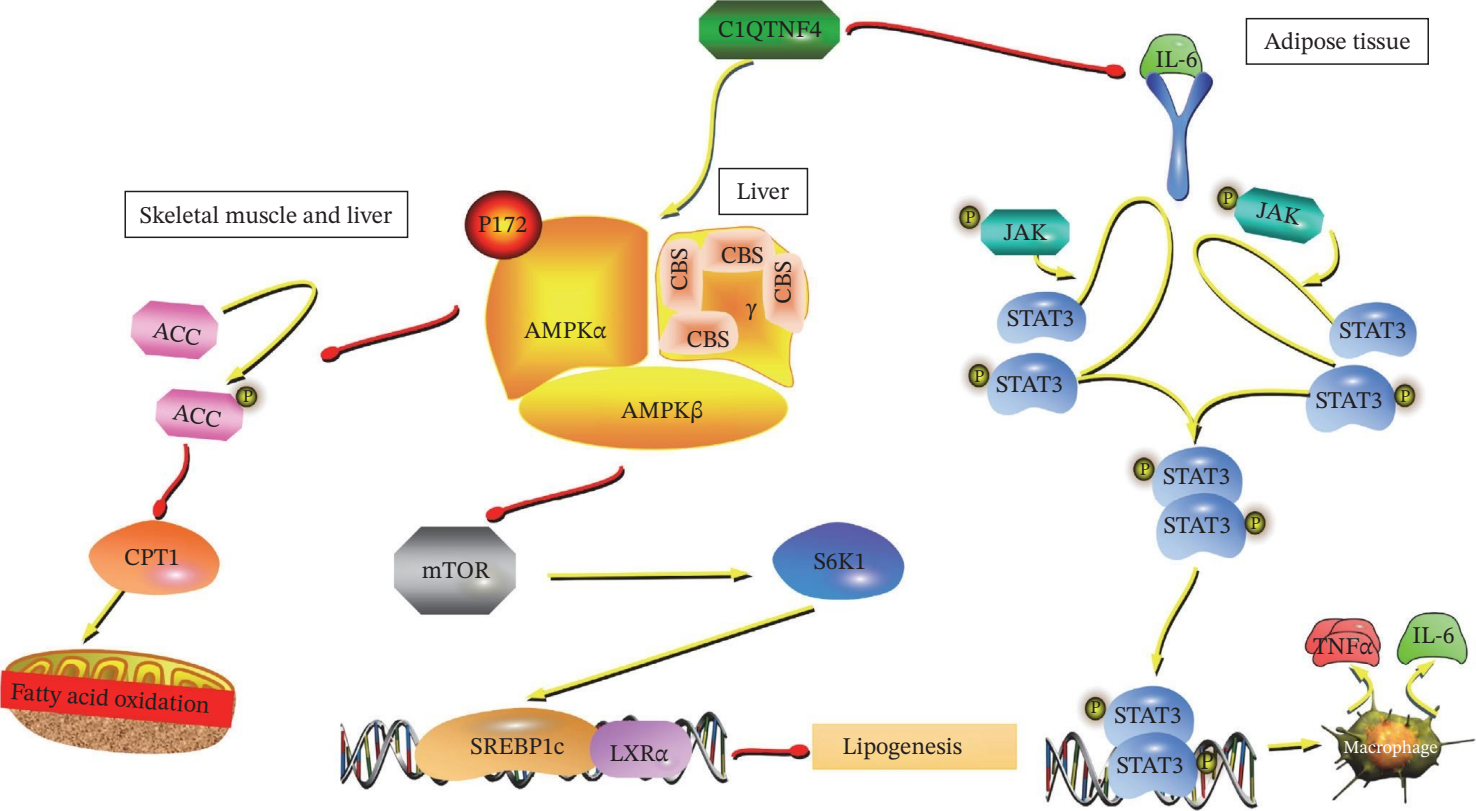
Putative mechanisms by which C1QTNF4 balances energy in skeletal muscle and liver tissue and alleviates inflammation in adipose tissue to treat diet‐induced obesity.

Our study proposes C1QTNF4 as a novel adipokine regulating lipid homeostasis to rectify energy imbalances and suppress inflammation. We demonstrated significantly elevated C1QTNF4 levels in diabetic patients compared to healthy individuals. Tg mice exhibited increased whole‐body energy expenditure, resistance to a HFD, heightened insulin sensitivity, and ameliorated adipose tissue inflammation. Further molecular analysis revealed tissue‐specific signaling pathways mediating these effects. In skeletal muscle, C1QTNF4 activated AMPKα to inhibit ACC, while in the liver, it activated AMPKα to inhibit S6K1/Srebp1c, ultimately promoting fatty acid oxidation for energy support and inhibiting lipogenesis to reduce lipid deposition. CLS infiltration in adipocytes and an altered IL‐6/JAK/STAT3 pathway in WAT of Tg mice was suggestive of C1QTNF4‐mediated inflammation suppression (Figure 8).

The observed elevation of C1QTNF4 in diabetic patients might be attributed to compensatory mechanisms within the body. Furthermore, in diabetic individuals with obesity, leptin levels were elevated, while adiponectin levels were decreased. Notably, leptin and adiponectin are prominent adipokines initially identified due to their altered expression in obesity and their regulatory role in adipocyte differentiation [21, 22]. While the atypical elevation of serum C1QTNF4 in diabetic patients appears contradictory to its established antidiabetic and antiobesity properties observed in Tg mice on a HFD, similar findings have been reported for C1QTNF1, with increased concentrations in type 2 diabetes mellitus (DM) patients despite its known protective function against nutritional challenges [23].

Previous research has documented the central role of C1QTNF4 in regulating food intake and body weight across diverse species, including humans, mice, and zebrafish [24]. Given the critical role of insulin sensitivity in metabolic regulation, the present study investigated the specific impact of C1QTNF4 on this important physiological process. Through a series of controlled experiments, we observed significant differences in food intake, VO_2_, and VCO_2_ between C1QTNF4 Tg mice and WT controls. These findings suggest a potential involvement of C1QTNF4 in modulating energy balance. Further examination via glucose and insulin tolerance tests revealed demonstrably enhanced insulin sensitivity in the Tg mice. These results provide compelling evidence for the direct activation of key energy balance pathways, including the insulin and energy expenditure pathways, by C1QTNF4. The observed ability of C1QTNF4 to simultaneously promote insulin sensitivity and suppress lipid deposition highlights its promising potential as a therapeutic target for ameliorating diabetic symptoms without the undesirable side effect of weight gain.

AMPK functions as an energy sensor that maintains cellular energy homeostasis [25]. It has been reported that C1QTNF1 decreases diet‐induced weight gain by enhancing fatty acid oxidation via AMPK activation and ACC inhibition to regulate lipid metabolism. Piperine can inhibit lipogenesis and treat hepatic steatosis by activating the adiponectin AMPK/S6K1/Srebp1c signaling pathway. C1QTNF4 suppresses its activation by suppressing the Shp2‐Ras‐ERK, JAK‐STAT3, and PI3K‐Akt/mTOR pathways [26]. Dysregulation of the JAK/STAT3 pathway may contribute to the development of obesity and diabetes [4]. Previously documented research has established the involvement of AMPK in lipid metabolism. Specifically, C1QTNF4 has been shown to: (1) activate AMPKα, leading to the inhibition of ACC and upregulation of CPT1 in skeletal muscle and liver tissue, thereby promoting fatty acid oxidation for energy production; (2) inhibit S6K1, thus downregulating Srebp1c in the liver and further propelling fatty acid oxidation; and (3) suppress hepatic de novo lipogenesis, reducing lipid deposition. Furthermore, established knowledge identifies IL‐6 as a mediator of proinflammatory activities, with the JAK/STAT signaling pathway critical for maintaining homeostasis. Prior studies have revealed that over 90% of macrophages within the adipose tissue of obese individuals cluster around dead adipocytes, suggesting that macrophage infiltration and the resultant chronic mild inflammation are causative factors in IR and, ultimately, diabetes. Interestingly, research has demonstrated the effectiveness of adipokines in mitigating chronic inflammation, highlighting the potential of anti‐inflammatory adipokines in addressing obesity‐associated metabolic and cardiovascular diseases. However, our novel findings demonstrate that C1QTNF4, while blocking the JAK/STAT3 pathway in WAT, also suppresses inflammation and infiltration of CLSs. Collectively, this evidence establishes C1QTNF4 as an adipokine regulating systemic energy balance and exerting anti‐inflammatory effects. We hypothesize that the observed amelioration of DIO is mediated by C1QTNF4’s regulation of lipid metabolism and obesity‐related inflammation. Moreover, this study represents the first demonstration of C1QTNF4’s metabolic regulatory role in peripheral organs. These findings suggest the immense potential of C1QTNF4 as a therapeutic target for obesity and diabetes.

## 4. Materials and Methods

### 4.1. Patients

A study conducted at Beijing Hospital recruited 48 participants aged 56–70 years (equally distributed across sexes) with varying health conditions, including obesity and DM. Participants were subsequently divided into two groups based on their health status. It is noteworthy that individuals with diabetes were not receiving any medication. Our study was carried out in accordance with the Code of Ethics of the World Medical Association (Declaration of Helsinki) for experiments involving humans. The study protocols were approved by the ethical review board of Beijing Hospital (Permit Number: LA2014110). Written informed consent was obtained from all subjects.

### 4.2. Generation of C1QTNF4 Tg Mice

Human C1QTNF4 Tg mice were established as described previously [27]. A C1QTNF4‐overexpressing vector in a pCAGGS backbone was first constructed and then microinjected into zygotes to establish founder Tg mice (on a C57BL/6J genetic background). The expression of the C1QTNF4 transgene was driven by the ubiquitous CAG promoter, which contains a CMV enhancer element with a chicken β‐actin promoter. F1 heterozygotes were generated by mating founder mice with WT mice, and C1QTNF4 Tg homozygotes were generated by mating the F1 littermates. The mouse line with detectable human C1QTNF4 protein in the serum was maintained and expanded for phenotypic analysis. No gross abnormalities were observed in these animals.

### 4.3. Animals

All the experimental procedures were performed according to the guidelines and policies approved by the Peking University Health Science Center. The mice were maintained in a specific pathogen‐free environment and had ad libitum access to food and water throughout the study period, except where noted. For the DIO model, C57BL/6 mice at 7 weeks of age (*n* = 32) weighing 20–25 g were divided into four groups of eight in both sexes. C1QTNF4 Tg mice and their corresponding littermate controls were given a high‐fat (HF; 60% kcal) diet (D12492; Research Diets, New Brunswick, NJ, USA) or a standard chow diet (4% fat SPF Rodent Feed, Beijing Keao Xieli Feed Co., Beijing, China) for 14 weeks. Food intake was measured for mice in individual cages every day over the study period.

### 4.4. Measurement of C1QTNF4 Protein in Serum and Plasma

The C1QTNF4 levels in serum and plasma were measured by flow cytometry using a cytometric bead assay, which was developed using BD^TM^ Cytometric Bead Array (CBA) products. An appropriate mouse antihuman C1QTNF4 monoclonal antibody was conjugated to the beads to prepare a stock of capture beads. Conjugation was performed following the instruction manual and with the reagents of a BD^TM^ CBA Functional Bead Conjugation Buffer Set (Cat. Number 558556, BD Bioscience‐PharMingen, San Diego, CA). The addition of a second fluorescent antibody (phycoerythrin [PE]‐labeled mouse antihuman C1QTNF4 monoclonal antibody) enabled the detection of the C1QTNF4 protein on the capture beads by flow cytometry. The assay was performed following the instruction manual and with the reagents of a BD^TM^ CBA Human Soluble Protein Master Buffer Kit (Cat. Number 558265, BD Bioscience‐PharMingen, San Diego, CA). Energy expenditure, respiratory quotient (RQ), oxygen consumption (VO_2_), carbon dioxide production (VCO_2_), locomotor activity, and food intake were measured using a comprehensive laboratory animal metabolic system (CLAMS; Columbus Instruments, Columbus, Ohio).

### 4.5. Body Composition

Body composition was examined with an Echo MRI (Echo Medical Systems, Houston, Texas) using a 3‐in‐1 Echo MRI Composition Analyzer.

### 4.6. Glucose and Insulin Tolerance Tests

Food was removed from the cages 16 h before the glucose tolerance testing (for fasting from 5 PM to 9 AM) or 2 h before insulin tolerance testing. Insulin was obtained from Sigma (St. Louis, MO, USA). After intraperitoneal injection (2 g/kg body weight glucose or 0.75 mIU/g body weight insulin), blood glucose was measured at 0, 15, 30, 60, and 120 min using a glucometer (ACCU‐CHEK, Roche, Basel, Switzerland).

### 4.7. Quantitative Real‐Time PCR (qRT‐PCR) Analysis

Total RNA was extracted from mouse tissues using TRIzol reagent (Carlsbad, CA, USA). First‐strand cDNA synthesis was performed using the RevertAid First Strand cDNA Synthesis Kit (Hudson, NH, USA). qRT‐PCR was conducted on an Opticon continuous fluorescence detection system (MJ Research Inc., Waltham, MA, USA) using SYBR green fluorescence (Molecular Probes, Eugene, OR, USA). Primer sequences for C1QTNF4, IL‐6, Srebf1 (SREBP1), JAK1, STAT3, S6K1, PRKAA1 (AMPKα1), ACC, and β‐actin are listed in Table 1. Relative gene expression was calculated using the comparative cycle threshold (CT) method, with β‐actin serving as the internal reference gene. Results were expressed as fold change relative to control samples.

**Table 1:** Primer sequences used for real‐time PCR.

Gene symbol	ENTREZ ID	Gene name	Forward primer	Reverse primer
C1QTNF4	67445	C1q and tumor necrosis factor‐related protein 4	GCTGCTCTTGCTGGGCTTCC	ACACCGTGTCGCCGTAGTCG
IL‐6	16193	Interleukin 6	TACCACTTCACAAGTCGGAGGC	TACCACTTCACAAGTCGGAGGC
Srebf1 (SREBP1)	20787	Sterol regulatory element binding transcription factor 1	CGACTACATCCGCTTCTTGCAG	CCTCCATAGACACATCTGTGCC
JAK1	16451	Janus kinase 1	CTGTCTACTCCATGAGCCAGCT	CCTCATCCTTGTAGTCCAGCAG
S6K1	72508	Ribosomal protein S6 kinase, polypeptide 1	AGGTGGAACCTCCCTTTAAGCC	CCAGAAAGACCTGGTTGGCACT
PRKAA1 (AMPK aIpha1)	105787	Protein kinase, AMP‐activated alpha 1 catalytic subunit	GGTGTACGGAAGGCAAAATGGC	CAGGATTCTTCCTTCGTACACGC
β‐actin	11461	Actb actin, beta	CGTGACATTAAGGAGAAGCTGTGC	CTCAGGAGGAGCAATGATCTTGAT
ACC	100705	Acetyl‐CoA carboxylase	AGAAGCGAGCACTGCAAGGTTG	GGAAGATGGACTCCACCTGGTT

### 4.8. Western Blot Analysis

Cells or tissues were lysed in cold isotonic lysis buffer (10 mM Tris‐HCl [pH 7.5], 0.5% Triton X‐100, and 150 mM NaCl with a complete protease inhibitor cocktail and PhosSTOP phosphatase inhibitor cocktail [Roche, Applied Science, Laval, PQ, Canada]) for 15 min on ice and were centrifuged for 15 min at 15,000 × g and 4°C. Then, the supernatant was collected and measured using a BCA protein quantification kit (Pierce, USA). The protein samples were separated by SDS–PAGE, and the separated proteins were electroblotted onto a nitrocellulose (NC) membrane (Whatman, Maidstone, United Kingdom). The membrane was then blocked and incubated with specific primary antibodies (shown in Table 2), followed by horseradish peroxidase‐labeled secondary antibodies for detection; chemiluminescence analysis was performed using an ECL chemiluminescence kit (Thermo Scientific, Etten‐Leur, The Netherlands). The C1QTNF4 polyclonal antibody was prepared as previously described [28], and the monoclonal antibodies were prepared by Beijing Protein Innovation. The other antibodies mentioned in this study were obtained from Cell Signaling Technology (Beverly, MA, USA).

**Table 2:** Antibody, isotype, company, product number, and MW of proteins.

Antibody	pAMPK	AMPK	pACC	ACC	pS6K1	S6K1	Srebp1c	IL‐6	pJAK	JAK	pSTAT3	STAT3	F4/80	β‐actin
Isotype	Rabbit IgG	Rabbit IgG	Rabbit IgG	Rabbit IgG	Rabbit IgG	Rabbit IgG	Mouse IgG	Rabbit IgG	Rabbit IgG	Rabbit IgG	Rabbit IgG	Rabbit IgG	Mouse IgG	Mouse IgG
Company	CST	CST	CST	CST	CST	CST	CST	CST	CST	CST	CST	CST	eBioscience	Sigma
Product number	2535	5831	3661	3662	9204	9202	9874	12912	3771	3230	9145	4904	15‐4801‐80	A1978
MW (kDa)	62	62	280	280	70, 85	70, 85	150	24	125	125	79, 86	79, 86	160	42

### 4.9. Histological Analysis

Tissues were fixed in PBS‐buffered 10% formalin for 2 days. After paraffin embedding, the tissue sections were stained with H&E using standard protocols. For immunohistochemistry, the tissue sections were deparaffinized and treated for antigen retrieval and blockade of endogenous peroxidase activity. After these processes, the tissue sections were stained with a primary F4/80 antibody and a biotinylated secondary antibody and were detected using a DAB kit (Dako, Glostrup, Denmark). The F4/80 antibody was obtained from eBioscience (clone BM8; San Diego, CA, USA). The slides were stained with hematoxylin after detection. For Oil Red O staining, frozen adipose tissue was embedded in an optimal cutting temperature (OCT) compound (Sakura, Tokyo, Japan). After fixing with 10% formalin, the adipose tissue sections were stained with Oil Red O (Sigma–Aldrich, St. Louis, MO, USA).

### 4.10. Measurements of Biochemical Molecules

The levels of glucose (Millipore, Billerica, MA, USA), adiponectin, and leptin (Linco Research, San Carlos, CA, USA) in mice were measured in duplicate using enzyme‐linked immunosorbent assay (ELISA) kits according to the manufacturer’s instructions. IL‐1, IL‐6, IL‐10, and TNFα levels were determined using an Aimplex Mouse Custom 4‐plex assay (T2C0411, QuantoBio, Beijing, China), which was performed by QuantoBio Co. Ltd. All experiments were repeated at least three times.

### 4.11. Detection of Liver Lipids

TC (esterified and free) and TGs in liver tissues were determined using enzymatic colorimetric assay kits purchased from Thermo Scientific (Middletown, USA). The liver tissues were collected from anesthetized animals. First, the abdomen and the thorax were opened. The circulation to the heart was cut off; after the animal was euthanized, the liver was dissected, weighed, and stored at −40°C for freeze‐drying. The lipids in the freeze‐dried livers were extracted in hexane and isopropanol (3:2 ratio) with 0.005% (v/w) 2,6‐di‐tert‐butyl‐4‐methylphenol, as previously described. The supernatants from the extraction were collected after centrifugation and dried under nitrogen flow at room temperature. The dried extracted lipids were redissolved in isopropanol containing 1% Triton X‐100 (v/v) and were then used for lipid determination.

### 4.12. Bioinformatics and Statistical Analysis

Statistical analyses were performed by the Kruskal–Wallis test or Mann–Whitney *U* test. The data were expressed as the means ± SEMs. *p* < 0.05 was statistically significant. Bioinformatics analysis was performed using DNAstar software.

## 5. Conclusions

Our research findings provide compelling evidence that C1QTNF4 functions as an adipokine, playing a critical role in regulating energy homeostasis and suppressing inflammation within an organism. We propose that the observed amelioration of DIO can be attributed to C1QTNF4’s modulatory effects on lipid metabolism and its ability to mitigate obesity‐associated inflammation. Furthermore, this study represents the first demonstration of C1QTNF4’s involvement in regulating metabolic processes within peripheral organs. These findings collectively highlight the immense potential of C1QTNF4 as a promising therapeutic target for the management of obesity and diabetes.

## Author Contributions

Conceptualization, validation: Daxiang Na, Lu Wang, and Hong Zhang. Methodology: Daxiang Na and Hong Zhang. Investigation: Daxiang Na. Formal analysis, data curation: Yanwei He. Resources: Xinrui Liu and Mingjun Huang. Writing – original draft preparation: Daxiang Na and Shuo Xie. Writing – review and editing: Xinrui Liu and Mingjun Huang. Visualization: Jingying Liu and Yanwei He. Supervision, project administration, funding acquisition: Lu Wang and Hong Zhang.

## Funding

This research was funded by the National Key R&D Program of China (Grant 2022YFA1104800), the National Natural Science Foundation of China (Grants 91129707 and 81172001), the Science and Technology Plan of Beijing City (Grant Z211100002921014), the Interdisciplinary Medicine Seed Fund of Peking University (Grant BMU2017MX003), and the Chinese Academy of Medical Sciences Innovation (Grant 2018‐I2M‐1‐002).

## Disclosure

All authors have read and agreed to the published version of the manuscript.

## Ethics Statement

The study was conducted according to the guidelines of the Declaration of Helsinki, and approved by the Biomedical Ethics Committee of Peking University (Protocol Codes LA2017036 and 2017‐03‐01).

## Consent

Informed consent was obtained from all subjects involved in the study.

## Conflicts of Interest

The authors declare no conflicts of interest.

## Supporting Information

Additional supporting information can be found online in the Supporting Information section.

## Supporting information


**Supporting Information** The following supporting information can be downloaded at www.mdpi.com/xxx/s1. Figure S1: Serum C1QTNF4 in normal males and females and diabetic obese individuals. Figure S2: (A–C) Homology analysis of C1QTNF4. (D–G) The fat mass, lean mass, and their proportions relative to body weight in four groups of mice. Figure S3: RT‐qPCR results for skeletal muscle, liver, and adipose tissue.

## Data Availability

Data are available upon reasonable request.
