# Supplementary material for: C1q/TNF‐Related Protein 4 (C1QTNF4) Acts as an Adipokine That Ameliorates Diet‐Induced Obesity by Improving Energy Metabolism and Alleviating Adipose Inflammation
Source: Mediators Inflamm. 2026 May 9;2026:7613074. doi: 10.1155/mi/7613074 (PMC13157307; doi:10.1155/mi/7613074)
Supplement: Supplementary file 1 — Supporting Information The following supporting information can be downloaded at www.mdpi.com/xxx/s1. Figure S1: Serum C1QTNF4 in normal males and females and diabetic obese individuals. Figure S2: (A–C) Homology analysis of C1QTNF4. (D–G) The fat mass, lean mass, and their proportions relative to body weight in four groups of mice. Figure S3: RT‐qPCR results for skeletal muscle, liver, and adipose tissue. [file MI-2026-7613074-s001.zip › supplementary.docx]

Supp.Figure1. Serum C1QTNF4 in healthy males and females and diabetic obese individuals: A. The expression level of C1QTNF4 in healthy female and male individuals B. The expression level of C1QTNF4 in obese or diabetic female and male individuals C. The expression level of C1QTNF4 in healthy and obese or diabetic male individuals D. The expression level of C1QTNF4 in healthy and obese or diabetic women.

Supp.Figure2. Homology analysis of C1QTNF4. A) Alignment of the C1q domains of C1QTNF4 family members. B) Identity of C1q domain protein sequences with the first C1q domain (left column) and the second C1q domain (right column) of C1QTNF4. C) Homology of C1QTNF4 in different species. D), E), F) &G) Fat mass, lean mass and their percentages of the whole-body weight.

Supp.Figure S3: RT-qPCR results for skeletal muscle,liver and adipose tissue.
