# Supplementary figures and images for: C1q/TNF‐Related Protein 4 (C1QTNF4) Acts as an Adipokine That Ameliorates Diet‐Induced Obesity by Improving Energy Metabolism and Alleviating Adipose Inflammation
Source: Mediators Inflamm. 2026 May 9;2026:7613074. doi: 10.1155/mi/7613074 (PMC13157307; doi:10.1155/mi/7613074)

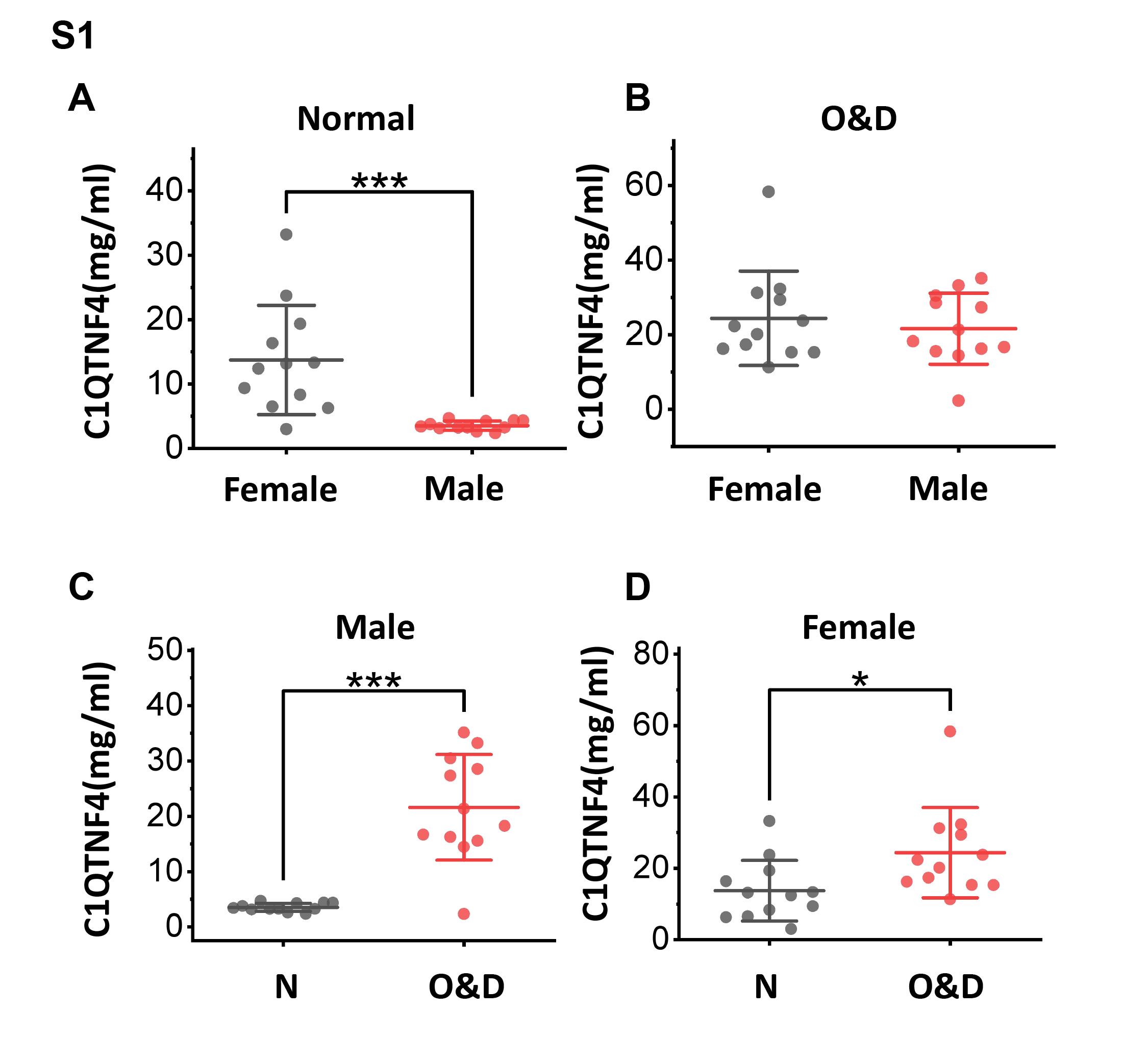

Supplement: Supplementary file 1 — Supporting Information The following supporting information can be downloaded at www.mdpi.com/xxx/s1. Figure S1: Serum C1QTNF4 in normal males and females and diabetic obese individuals. Figure S2: (A–C) Homology analysis of C1QTNF4. (D–G) The fat mass, lean mass, and their proportions relative to body weight in four groups of mice. Figure S3: RT‐qPCR results for skeletal muscle, liver, and adipose tissue. [file MI-2026-7613074-s001.zip › S1.tif]

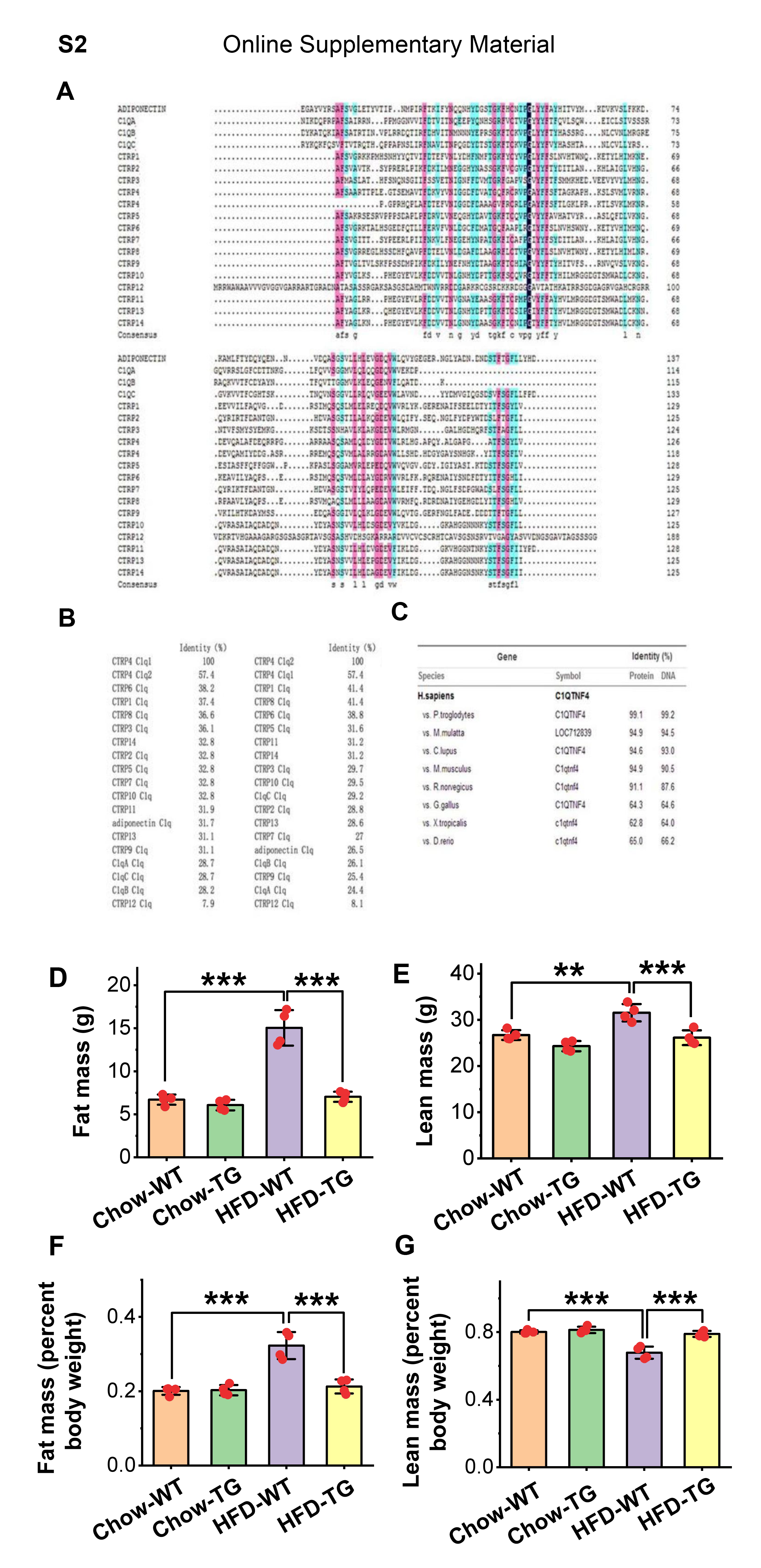

Supplement: Supplementary file 1 — Supporting Information The following supporting information can be downloaded at www.mdpi.com/xxx/s1. Figure S1: Serum C1QTNF4 in normal males and females and diabetic obese individuals. Figure S2: (A–C) Homology analysis of C1QTNF4. (D–G) The fat mass, lean mass, and their proportions relative to body weight in four groups of mice. Figure S3: RT‐qPCR results for skeletal muscle, liver, and adipose tissue. [file MI-2026-7613074-s001.zip › S2.tif]

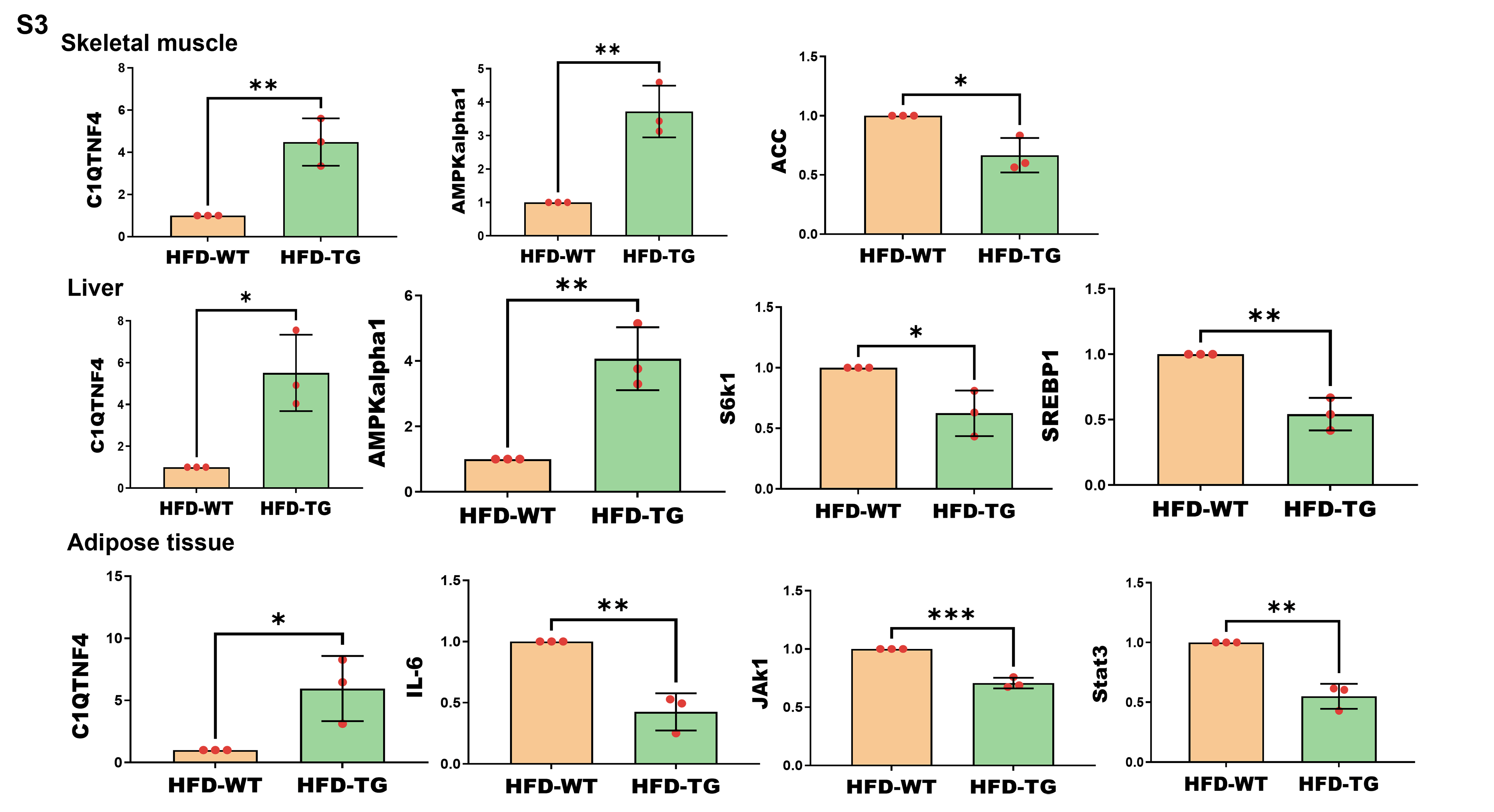

Supplement: Supplementary file 1 — Supporting Information The following supporting information can be downloaded at www.mdpi.com/xxx/s1. Figure S1: Serum C1QTNF4 in normal males and females and diabetic obese individuals. Figure S2: (A–C) Homology analysis of C1QTNF4. (D–G) The fat mass, lean mass, and their proportions relative to body weight in four groups of mice. Figure S3: RT‐qPCR results for skeletal muscle, liver, and adipose tissue. [file MI-2026-7613074-s001.zip › S3.tif]
